# Mammalian TIMELESS Is Involved in Period Determination and DNA Damage-Dependent Phase Advancing of the Circadian Clock

**DOI:** 10.1371/journal.pone.0056623

**Published:** 2013-02-13

**Authors:** Erik Engelen, Roel C. Janssens, Kazuhiro Yagita, Veronique A. J. Smits, Gijsbertus T. J. van der Horst, Filippo Tamanini

**Affiliations:** 1 Department of Genetics, Center for Biomedical Genetics, Erasmus University Medical Center, Rotterdam, The Netherlands; 2 Department of Cell Biology, Center for Biomedical Genetics, Erasmus University Medical Center, Rotterdam, The Netherlands; 3 Department of Neuroscience and Cell Biology, Kyoto Prefectural University of Medicine, Kyoto, Japan; 4 Unidad de Investigación, Hospital Universitario de Canarias, Instituto de Tecnologias Biomedicas, Tenerife, Spain; CNRS UMR7275, France

## Abstract

The transcription/translation feedback loop-based molecular oscillator underlying the generation of circadian gene expression is preserved in almost all organisms. Interestingly, the animal circadian clock proteins CRYPTOCHROME (CRY), PERIOD (PER) and TIMELESS (TIM) are strongly conserved at the amino acid level through evolution. Within this evolutionary frame, TIM represents a fascinating puzzle. While *Drosophila* contains two paralogs, dTIM and dTIM2, acting in clock/photoreception and chromosome integrity/photoreception respectively, mammals contain only one TIM homolog. Whereas TIM has been shown to regulate replication termination and cell cycle progression, its functional link to the circadian clock is under debate. Here we show that RNAi-mediated knockdown of TIM in NIH3T3 and U2OS cells shortens the period by 1 hour and diminishes DNA damage-dependent phase advancing. Furthermore, we reveal that the N-terminus of TIM is sufficient for interaction with CRY1 and CHK1 as well for homodimerization, and the C-terminus is necessary for nuclear localization. Interestingly, the long TIM isoform (l-TIM), but not the short (s-TIM), interacts with CRY1 and both proteins can reciprocally regulate their nuclear translocation in transiently transfected COS7 cells. Finally, we demonstrate that co-expression of PER2 abolishes the formation of the TIM/CRY1 complex through affinity binding competition to the C-terminal tail of CRY1. Notably, the presence of the latter protein region evolutionarily and structurally distinguishes mammalian from insect CRYs. We propose that the dynamic interaction between these three proteins could represent a post-translational aspect of the mammalian circadian clock that is important for its pace and adaption to external stimuli, such as DNA damage and/or light.

## Introduction

In mammals, the circadian system is composed of a central circadian pacemaker in the suprachiasmatic nuclei (SCN) of the brain and peripheral oscillators in virtually any other cell and organ. To remain synchronized with the day-night cycle, the SCN clock is daily reset by light information (photoentrainment), detected by retinal photoreceptors in the eye that stimulate the neurons in the SCN via the retinohypothalamic tract [1]. In turn, the SCN synchronizes the intracellular clocks in peripheral organs through direct innervations and the release of humoral factors.

Molecular and genetic analyses of the circadian clock in plants (*Arabidopsis*), fungi (*Neurospora*), insects (*Drosophila*), amphibia (*Xenopus*), fish (zebrafish), and mammals (mouse/humans) have revealed that circadian rhythms are generated by a molecular oscillator. This has been hypothesized to consist of auto-regulatory positive and negative transcription-translation feedback loops in which cyclically expressed clock gene products regulate their own expression with an approximate 24-h period [2]. In the positive loop of the mammalian molecular oscillator, the transcription factor Brain and muscle Arnt-like protein-1 (BMAL1) heterodimerizes with either Circadian Locomotor Output Cycles Kaput (CLOCK) or Neuronal PAS domain protein 2 (NPAS2) and drives transcription of the Cryptochrome (*Cry1* and *Cry2*) and Period (*Per1* and *Per2*) genes through E-box enhancer elements in their promoters. After a delay of several hours, the gene products accumulate and form CRY/PER heterodimers that accumulate in the nucleus and shut down their own expression (negative feedback) by inhibiting CLOCK-BMAL1 mediated transcription [3], [4], [5]. Inactivation of *Bmal1* [6] or simultaneous inactivation of *Cry1* and *Cry2* [7] results in an immediate loss of rhythmicity at the behavioral and molecular level, demonstrating the importance of these positive and negative feedback loops. In addition, prominent post-translational modification of clock proteins occurs [8]. Particularly, regulated phosphorylation and ubiquitination of the PER and CRY proteins (determining the rate of degradation, and successive accumulation of these proteins) and signal-mediated sub-cellular localization of these protein complexes are important in establishing the delay in *Cry* and *Per* mRNA and protein peaks [9], [10].

Interestingly, several studies have shown that the cell cycle [11] as well as the DNA damage response (DDR; including cell cycle checkpoint activation and DNA repair) upon exposure to genotoxic stress [12], [13], are connected to the circadian clock. We and others have shown that the connection between the mammalian clock and the DDR is reciprocal and presumably evolutionarily conserved, as genotoxic agents can phase advance the molecular oscillator in a circadian phase and dose dependent manner in *Neurospora,* rat and human cells, as well as in the living mouse [14], [15]. In mammals, DNA damage-induced phase shifting was shown to require ATM/ATR and NBS damage signaling [14].

The mammalian TIMELESS (TIM) protein, originally identified based on its similarity to *Drosophila* dTIM [16], [17], interacts with the clock proteins dCRY and dPER and is essential for circadian rhythm generation and photo-entrainment in the fly [18]. However, recent phylogenetic sequence analysis has demonstrated that TIM is not the true ortholog of dTIM, but rather shares (even greater) similarity to a second family of proteins that are more widely conserved in eukaryotes [19]. These include *Drosophila* dTIM-2 (paraloge of dTIM), *Saccharomyces cerevisiae* Tof1p, *Schizosaccharomyces pombe* Swi1p, and *Caenorhabditis elegans* TIM. With the exception of dTIM-2, that has an additional function in retinal photoreception [20], these proteins are not involved in the core clock mechanism, but instead are at the heart of molecular pathways important for chromosome integrity, efficient cell growth and/or development. Consistently, knockout of the mouse *Tim* gene results in embryonic lethality just after blastocyst implantation [21], while Q1008E and A429D missense mutations in *hTIM* have been identified as candidate “drivers” in breast cancer [22]. Intriguingly, down-regulation of mammalian *Tim* by RNA interference (RNAi) not only disrupts the ATM/ATR signaling and DNA replication pathways in cultured cells [23], [24], [25], but also electrical circadian rhythm in mouse SCN slices [26], suggesting that this protein may have acquired a dual function in mammals. The above concept is re-enforced by the observed *in vitro* physical interactions of TIM with both CRYs and CHK1, a checkpoint kinase activated by ATR [23], [27]. Despite the important role of mammalian TIM in biological processes such as DNA replication, ATM/ATR signaling, and circadian rhythm generation, insight into how TIM protein domains contribute to these processes is lacking.

The deregulation of both cell cycle and circadian clock is implicated in cancer aetiology [12]. Since TIM functionally intersects with the above two processes, we decided to perform a structure-function study of this protein and analyze its contribution to the clock functioning in proliferative tissues. Our study reveals that the extreme C-terminus of TIM is responsible for its nuclear localization and that self-dimerization occurs in the N-terminus. Moreover, we demonstrate a primary role for TIM in setting the period of peripheral clocks and show how the mammalian TIM-CRY-PER complex mechanistically and evolutionary differs from that of *Drosophila*.

## Results

### Identification of the protein regions involved in nuclear localization and self-dimerization of TIM

**Figure 1:**
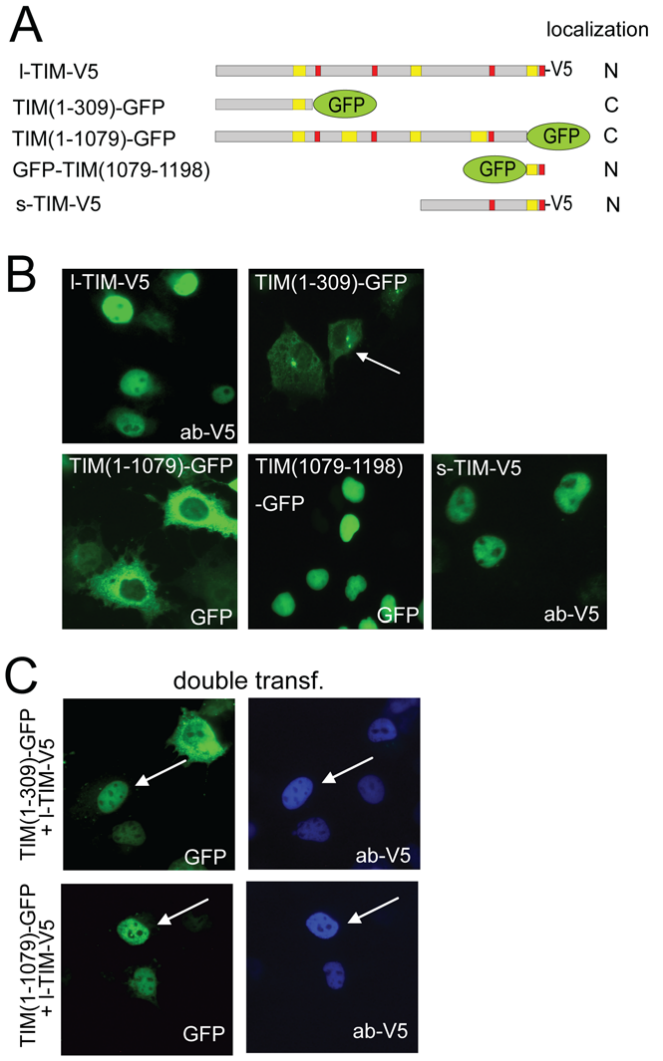
Identfication of protein domains involved in nuclear localization and dimerization of TIM. **A**) Schematic diagram of the V5-tagged long (l-full length) and short TIM proteins (s), as well as GFP-tagged truncated versions, including the position of the putative nuclear localization signals (red) and coiled-coil domains (yellow). Subcellular localization, as observed during the experiments is represented by N (nuclear) or C (cytoplasmic). **B**) Representative examples of the subcellular distribution patterns of the various tagged TIM proteins in COS7 cells, as detected by GFP fluorescence or staining with anti-V5 antibodies. Note that TIM(1–309)-GFP appears to be subject to accelerated proteolytic degradation, as it is expressed at lower level and shows signs of accumulation at the aggresome (arrow), a cytoplasmic structure involved in 26S proteasome degradation. **C**) Representative examples of COS7 cells transiently expressing truncated TIM(1–1079)-GFP or TIM(1–309)-GFP proteins (green) together with l-TIM-V5 (blue). Arrows show truncated TIM-GFP proteins that are translocated to the nucleus in presence of l-TIM-V5.

Since both endogenous and over-expressed TIM have been detected exclusively in the nucleoplasm [27], we initially focused on the domains that could determine its cellular localization. Through visual inspection of the full length mouse protein sequence (amino acids 1 to 1198), we identified 4 putative nuclear localization signals (NLS), located at amino acid position 316–322 (NLS1, RRVPKRR), 528–537 (NLS2, KRKKRKKKKK), 935–946 (NLS3, RRQLYKKRRKK), and 1175–1190 (NLS4, RRN_10_ RKKR). These NLS sequences are fully conserved in the human TIM protein (data not shown). To investigate whether the above domains are functionally relevant, we generated a panel of full length and truncated green fluorescent protein (GFP)-tagged or V5-tagged mouse TIM expression constructs (Fig. 1A) and analyzed the sub-cellular distribution of these tagged TIM proteins in transiently transfected COS7 cells. As expected, C-terminally V5 tagged full length mouse TIM (hereafter called l-TIM-V5), was detected in the nucleus with exclusion of the nucleoli (Fig. 1B). Instead, the localization of TIM(1–309)-GFP as well as TIM(1–1079)-GFP was cytoplasmic (Fig. 1B), indicating a possible involvement of the C-terminus in nuclear import. Indeed, despite the fact that its molecular weight (∼45 kDa) would allow free cellular diffusion between the cytoplasm and the nucleus, GFP-TIM(1079–1198) fully localizes to the nucleoplasm as well as the nucleolus (Fig. 1B). From these data we conclude that the C-terminal NLS4 is likely the relevant nuclear import driver of TIM (although its mutagenesis would give the definitive answer) and that yet unidentified protein domains in the remainder of the protein are necessary for its nucleolar exclusion.

In addition to full length TIM (∼140 KDa), a shorter C-terminal isoform (s-TIM) with yet unknown function was found to be expressed at constitutively high levels in brain SCN slices [26]. Although it is unknown whether s-TIM originates from an additional transcription initiation site, or from alternative splicing, Western blot analysis using C-terminal anti-TIM antibodies has revealed that s-TIM has a size of ∼50 KDa and may corresponds to the last 475 amino acids of the protein [26]. We mapped *in silico* two potential starting methionines for s-TIM at amino acid position 722 (ATG1) and 732 (ATG2), and using a suitable restriction enzyme (see material and methods), we generated an expression construct for TIM(732–1198)-V5 starting from the ATG2 (hereafter called s-TIM-V5) (Fig. 1A). Interestingly, we observed that s-TIM-V5 was well expressed and localizes into the nucleus and is excluded from the nucleoli, similar to l-TIM-V5 (Fig. 1B). This result supports the above mapping of the functional NLS of TIM within its extreme C-terminus and suggests that sequences preventing the protein to enter the nucleoli are localized between aa 732 and 1079.

TIM has been reported to form homo-multimeric complexes *in vitro*, while its binding partner TIPIN disrupts TIM self-association [27]. To further expand these results, we investigated whether TIM is able to self-associate in living cells. COS7 cells were co-transfected with l-TIM-V5 and TIM(1–1079)-GFP, proteins that are observed in the nucleus and cytoplasm, respectively, when individually expressed. Interestingly, TIM(1–1079)-GFP became readily nuclear in the presence of l-TIM-V5 (Fig. 1C), thereby suggesting that TIM is able to self-associate in a cellular context. It is possible that the responsible domain for this dimerization is localized at the N-terminus of TIM, as TIM(1–309)-GFP (individually cytoplasmic) was also efficiently translocated to the nucleus by l-TIM-V5 (Fig. 1C).

### TIM downregulation shortens the circadian period in cultured cells

**Figure 2:**
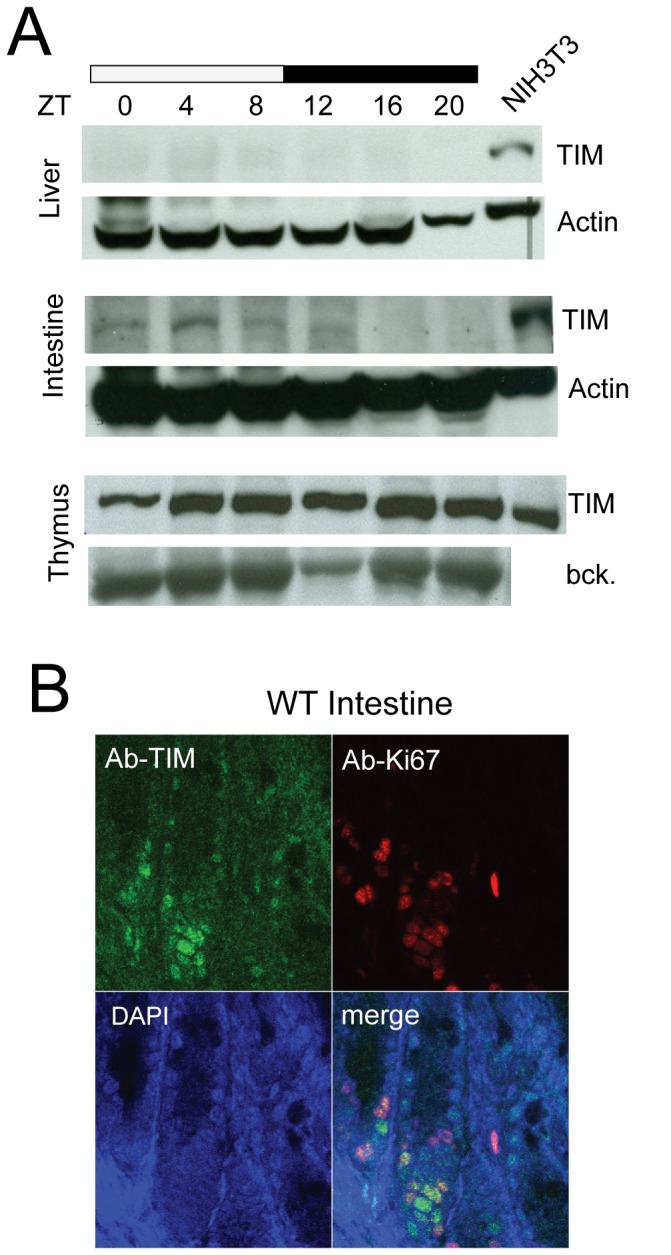
Protein analysis of TIM in wild type mouse tissues collected in a circadian fashion. **A**) Western blot analysis of temporal TIM expression in liver (top), intestine (middle) and thymus (bottom) from wild type mice housed under a LD12∶12 light regime and sacrificed every 4 hours. The filter was probed with anti-TIM antibodies (kindly provided by P. Minoo [37]) and β-Actin immunostaining served as a loading control. In the case of thymus a background band was used as internal control (bck.) On each blot protein lysates of NIH3T3 cells was loaded as positive control for TIM immunostainig procedure. **B**) Immunofluorescence picture of the mouse intestine. TIM was immunostained with anti-TIM (green) and proliferative cells were visualized by K67 staining (red). Note that TIM expression is confined to the proliferative compartment of the intestinal villi (crypt) and not always overlaps with K67 staining.

RNAi-mediated down-regulation of TIM in mouse SCN slices caused total loss of circadian electrical activity, which is a well known circadian output. This observation led to the concept that TIM is an essential component of the clock mechanism, but could not be confirmed *in vivo* as Tim KO mice are embryonic lethal [21],

During this work we observed that TIM protein was predominantly and robustly expressed in proliferative organs (spleen, thymus, intestine and testis) compared to those more differentiated such as kidney and liver (Fig. S1A), which is in good agreement with its reported mRNA expression patterns [17]. Next, we examined whether TIM expression could undergo daily variation in liver, intestine and thymus of adult wild type mice housed under a regular (LD12∶12) light regime (Fig. 2 A). Whereas we could not detect TIM in liver, TIM showed a circadian expression pattern in the intestine with peaks at ZT 4 and ZT8. Immuno-histochemical staining of cryostat sections of the intestine revealed that TIM is exclusively present in the nuclei of cells present at the bottom of the crypts, which represent the proliferative compartment of this organ (Fig. 2B). In the thymus TIM was expressed at constitutive level through out the circadian cycle.

**Figure 3:**
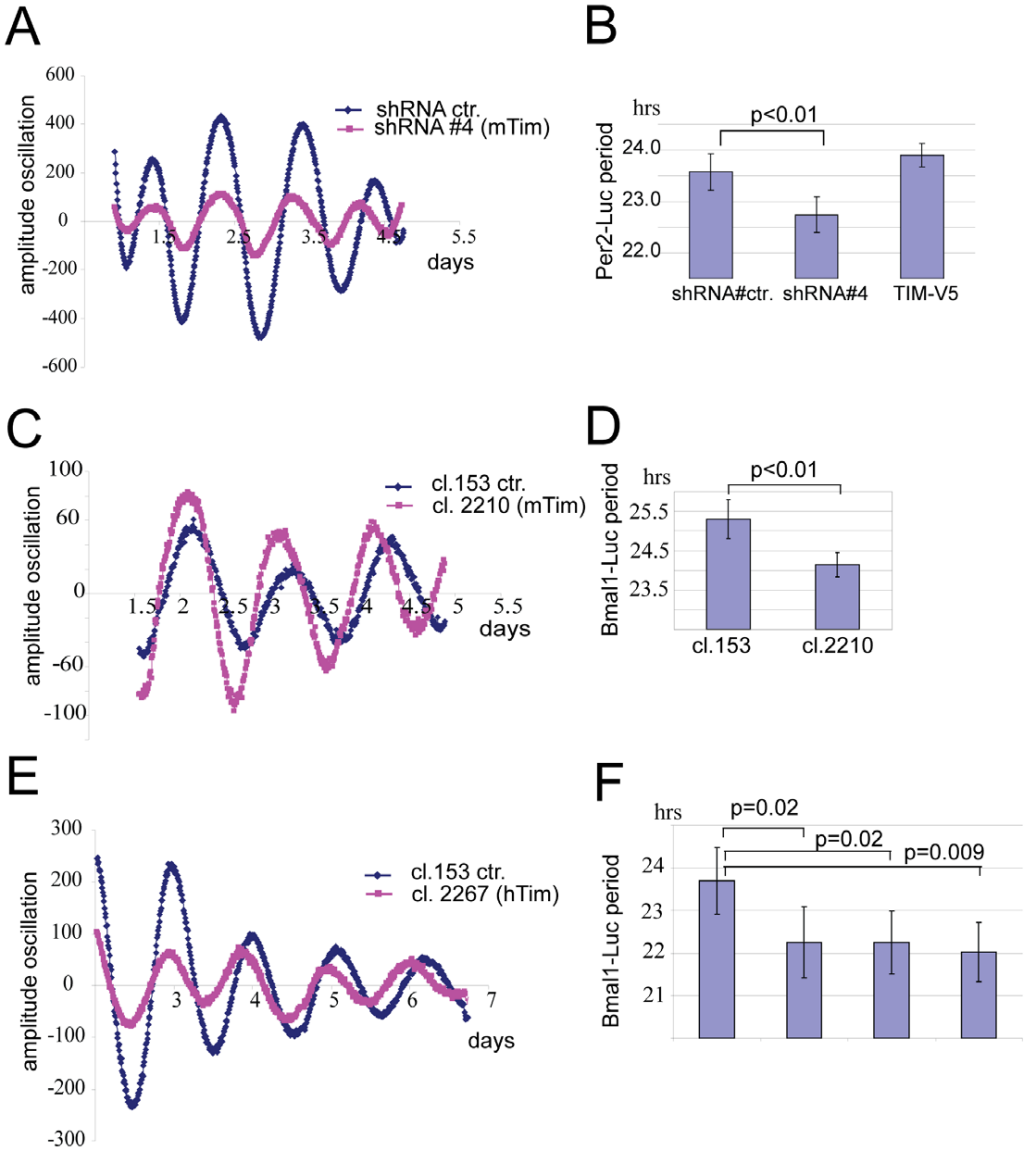
RNAi knockdown of mouse and human TIM shortens the period in cultured cells. **A**) Example of bioluminescence oscillations in Forskolin-synchronized NIH 3T3 cells co-transfected with the mPer2:Luc clock reporter and shRNActr (blue), or shRNA#4 (pink). Data are presented after a 24 moving average correction and base line correction. **B**) Quantification of the period of oscillations shown in panel A, including the period after over-expression of l-TIM-V5. **C**) Example of bioluminescence oscillations of Forskolin-synchronized NIH 3T3 cells co-transfected with Bmal1:Luc reporter and shRNA153 (control), or shRNA2210 (against mouse TIM). **D**) Quantification of the cellular period of oscillations shown in panel C. **E**) Example of bioluminescence oscillations in Forskolin-synchronized U2OS cells co-transfected with mBmal1:Luc reporter and shRNA153 (control), or shRNA2267 (against human TIM). **F**) Quantification of the period of oscillations for shRNA153 and shRNA2267 shown in panel E, including other 2 independent shRNA constructs against hTIM causing similar period shortening (shRNA2268 and shRNA2270). All data presented are average+−SD of at least 4 independent experiments. The significance of shortening of period from *Tim* knockdown was calculated wit a t-test (1-tailed distribution, two-sample unequal variance).Three oscillation peaks were taking into the calculation.

Since the use of cultured cells is an established system to test the functionality of the clock at the cell autonomous level [28], we performed RNAi-mediated down-regulation of TIM in cultured cells, in which the protein is abundantly expressed. For this we generated 4 shRNA vectors (#1 to #4) directed against the mouse *Tim* sequence. Western blot as well as immuno-fluorescence analysis of NIH3T3 cells transfected with these plasmids showed that we successfully reduced the expression of endogenous TIM with shRNA#4 (Fig. S1B and 1D, respectively), and its efficiency was further confirmed by analyzing protein lysates derived from HEK293 cells transiently co-transfected with l-TIM-V5 and shRNA#4 (Fig. S1C). Next, we co-transfected shRNA#4 with the clock reporter *Per2*-Luciferase in NIH 3T3 cells and analyzed clock performance in real time after an initial clock synchronization with Forskolin (Fig. 3A). Interestingly, down-regulation of TIM, but not its over-expression with l-TIM-V5, caused a significant (p<0.01) shortening of the period of about 1 hour (22.7 hrs±0.3 hrs) compared to the control (23.6 hrs±0.4 hrs) (Fig. 3B). By using a different shRNA construct against mouse *Tim* (clone 2210, which was previously validated in [29]), we again observed a 1 hour shortening of the period in NIH 3T3 cells (Fig. 3E/F, control shRNA153 25.3 hrs±0.48 hrs, shRNA2210 24.15 hrs±0.31 hrs, p<0.01) (Fig. 3C and 3D).

Since RNAi down-regulation of other clock modifiers (eg. *Bmal2*) has produced some inconsistent results between mouse [30] and human cells [31], we then asked whether down-regulation of TIM could cause a shortening of the circadian period in human cells. U2OS cells were co-transfected with *Bmal1*-Luc and 3 independent shRNA vectors targeting the human *Tim* sequence. Successful down-regulation of *hTim* mRNA with these shRNA constructs was verified by qPCR (Figure S1E). As shown in Fig. 3E and 3F, down-regulation of human TIM caused a statistically significant shortening of the cellular period by at least 1 hour, as compared to U2OS cells expressing non targeting control shRNAs (clone 153).

In conclusion, these results support a role for TIM in determining the periodicity of the peripheral oscillator, and suggest its possible different contributions to the clock mechanism in SCN and cultured cells.

### Down-regulation of TIM diminishes DNA damage-dependent phase advance

**Figure 4:**
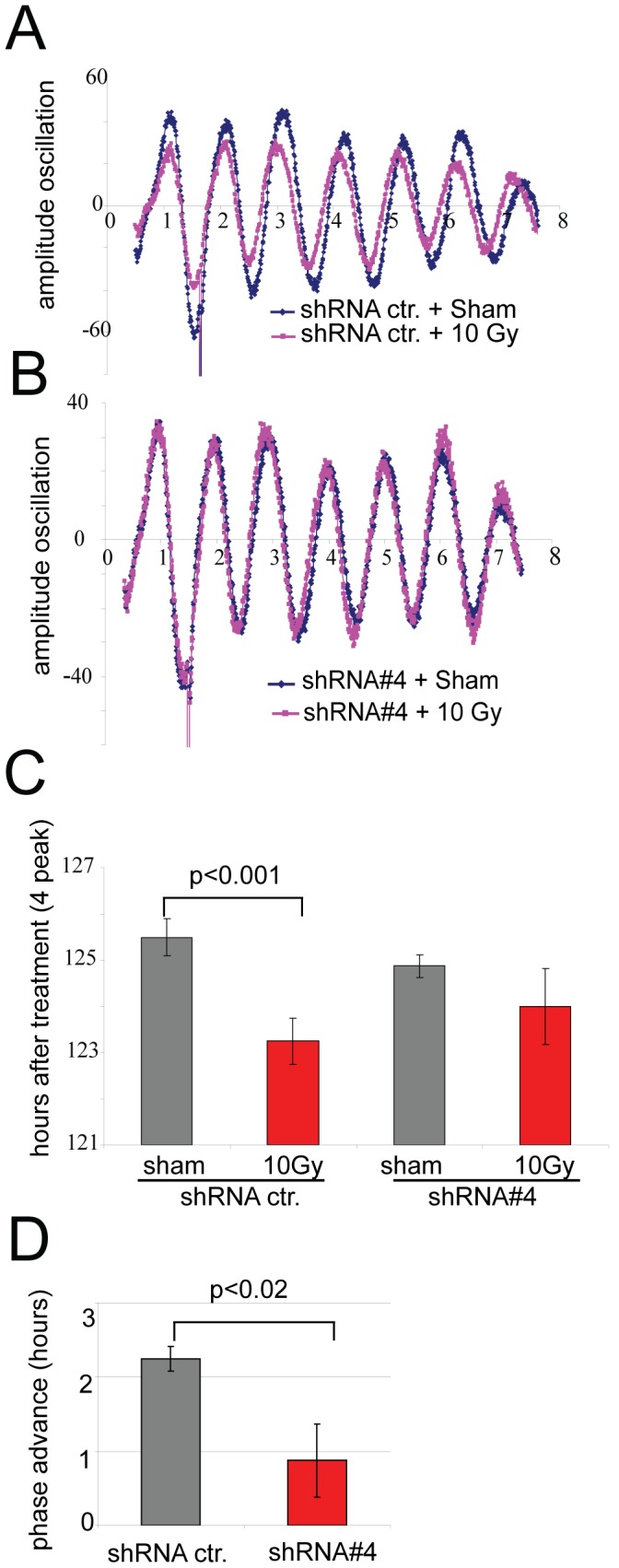
TIM downregulation attenuates DNA-damage dependent phase advance. **A**) Bioluminescence oscillations of forskolin-synchronized NIH 3T3 cells co-transfected with a Bmal1:Luc reporter and shRNActr. After the first peak (1.5 day after synchronization) dishes were subjected to 10 Gy irradiation (pink) or mock treated (blue) and bioluminescence rythms were followed for another 6 days. **B**) NIH3T3 cells were co-transfected with Bmal1-Luc and shRNAi#4 against mTIM and similarly exposed to 10J irradiation. **C**) Quantification of the ionizing radiation induced clock phase advance, shown in panel A and B. For that we calculated the time necessary to reach the 4 peak in presence of shRNActr/sham (125 hrs+/−0.4 hrs std), shRNActr/10 Gy (123.25 hrs+/−0.5 hrs std), shRNA#4/sham (124.8 hrs+/−0.25 hrs std), and shRNA#4/10 Gy (124 hrs+/−0.81 hrs stdev).**D**) Quantitative analysis of the magnitude of ionizing radiation-induced phase advances in shRNActr. and shRNA#4. The overall mean values are shown. Error bars represent StDev, and significance was calculated with a t-test.

We have previously shown that ionizing radiation (IR) and other genotoxic agents can phase-advance the circadian clock in a dose- and time-dependent manner [14]. Noteworthy, IR-induced phase shifting of the mammalian circadian clock relies on a functional ATM and does neither involve temporary changes in the expression level of known clock genes (i.e. *Per1*, *Per2*, *Cry1*, *Cry2*, *Bmal1*, *Clock*), nor *de novo* protein synthesis, suggesting that some post-translational modification mechanism might be at work. Because TIM is functionally and physically connected to both ATM and ATR signaling pathways [25], [29], we next investigated the possible involvement of TIM in DNA damage-mediated phase shifting of the clock. To this end, we knocked down TIM in confluent NIH 3T3 cells using the plasmid shRNA#4 that was co-transfected with the Bmal1-Luc reporter. At 28 hours after clock synchronization with Forskolin, cells were exposed to either 10 Gy, or a mock treatment (Fig. 4A for control shRNA, Fig 4B for shRNA#4). Interestingly, whereas cells co-transfected with a control shRNA showed the expected ionizing radiation-induced phase advance of ∼2 hour, down-regulation of TIM using shRNA#4 significantly diminished this effect (Fig. 4C and Fig. 4D, p<0.02). From these data we conclude that TIM is required for DNA damage induced phase shifting of the clock, and as such may play an important role in connecting the DNA damage response and circadian clock machineries.

### Mapping the regions involved in the association between TIM/CRY1 and TIM/CHK1

Previously, physical interaction of TIM with the core clock components CRY1 and CRY2 has been reported by over-expressing these proteins in HEK293 cells [23], [27]. To investigate whether such interactions also occur under semi-physiological conditions, we have used NIH 3T3^HA-CRY1^ cells, which express wild type HA-CRY1 at constitutively low level (Fig. S2A). Pull down of HA-CRY1 from NIH 3T3^HA-CRY1^ cell lysates resulted in specific co-precipitation of endogenous TIM (Fig. S2B). This finding demonstrates that the interaction of TIM with the clock machinery occurs also at semi-physiological level.

**Figure 5:**
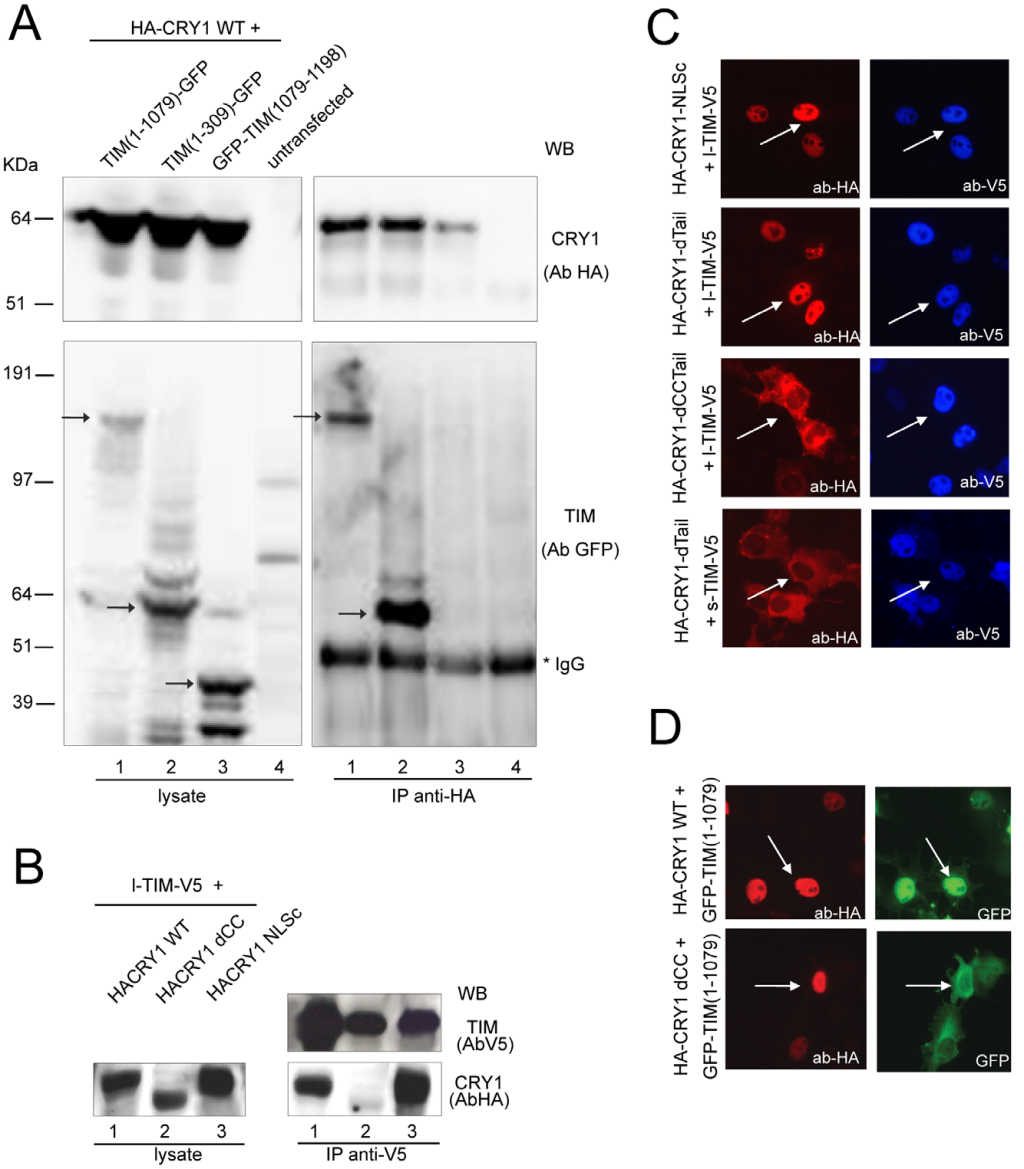
The C-terminal coil-coiled of mCRY1 interacts with the N-terminus of TIM, thereby promoting mutual nuclear accumulation. **A**) Identification of TIM regions engaged in the interaction with CRY1. HA-CRY1 WT was immunoprecipitated from cell lysates of COS7 cells transiently co-expressing HA-CRY WT and various truncated GFP-tagged TIM proteins. The input (lysate) and immunpoprecipitate (IP anti-HA) were analyzed for the presence of TIM using anti-GFP antibodies. **B**) Identification of CRY1 regions engaged in the interaction with TIM. TIM was immunoprecipitated from cell lysates of COS7 cells transiently co-expressing l-TIM-V5 and wild type HA-CRY1, HA-CRY1ΔNLSc or HA-CRY1ΔCC. The input (lysate) and immunoprecipitate (IP anti-V5) were analyzed for the presence of HA-CRY1 proteins using anti-HA antibodies. **C**) Subcellular localization of mutant HA-CRY1 proteins in COS7 cells in the absence (left panels) or presence (right panels) of l-TIM-V5 as detected with anti-HA (CRY1, red) and anti-V5 (TIM, blue) antibodies. Representative examples of fluorescent cells are shown. **D**) Subcellular localization of truncated TIM(1–1079)-GFP (green) in COS7 cells co-expressing HA-CRY1 WT (top, red), or HA-CRY1dCC) (bottom, red).

Next, we determined the TIM and CRY1 protein regions involved in this association. Mutant TIM proteins were transiently co-expressed with HA-CRY1 WT (wild type) in COS7 cells and the corresponding lysates were subjected to immunoprecipition with anti-HA antibodies. As shown in Fig. 5A, TIM(1–1079)-GFP and TIM(1–309)-GFP, but not TIM(1079–1198)-GFP, co-precipitate with HA-CRY1, thereby revealing that the extreme N-terminus of TIM (aa 1–309) is sufficient for this association. Since CHK1, a regulator of the ATR-dependent DNA damage response, is another known partner of TIM, we similarly tested their mode of interaction in transfected COS7 cells. Interestingly, the N-terminal region of TIM (1–309) is also sufficient for binding to Flag-CHK1 or to some endogenous proteins that are in complex with it (Fig. S2C).

To define the region of CRY1 involved in association with TIM, we used a previously characterized panel of HA-CRY1 mutant proteins which either lacked the C-terminal coiled-coil domain (HA-CRY1dCC), the C-terminal tail (HA-CRY1dTail), or both (HA-CRY1dCCtail), or which contain a mutated C-terminal NLS (HA-CRY1mutNLS_C_) [32]. As shown in Fig. 5B, HA-CRY1 WT and HA-CRY1mutNLSc were efficiently co-immunoprecipated together with l-TIM-V5, whereas HA-CRY1dCC failed to do so. Similarly, HA-CRY1dtail, but not HA-CRY1dCCtail, co-immunoprecipitated with l-TIM-V5 (data not shown). This result indicates that the C-terminal coiled-coil domain of CRY1 is involved in the association with TIM. As the C-terminus of CRY2 carries a coiled-coil domain in a similar position (Fig. S4), it is likely that CRY2 interacts with TIM through its C-terminal coiled-coil.

Next we investigated the effect of TIM-CRY1 interactions on their sub-cellular localizations. As shown previously [32], upon over-expression in COS7 cells, HA-CRY1mutNLSc shows a nucleo-cytoplasmic localization, whereas HA-CRY1dtail and HA-CRY1dCCtail are mainly cytoplasmic. Interestingly, co-expression with l-TIM-V5 renders HA-CRY1mutNLSc and HA-CRY1dtail completely nuclear. In contrast, HA-CRY1dCCtail remained in the cytoplasm in the presence of l-TIM-V5 (Fig. 5C). In a similar set of experiments, we observed that, in contrast to l-TIM-V5, s-TIM-V5 was not able to translocate HA-CRY1dtail (Fig. 5C) and HA-CRY1mutNLS (data not shown) into the nucleus, which is in complete agreement with our observation that s-TIM-V5 lacks the N-terminal domains (aa 1–309) necessary for interaction with CRY1. Finally, to further corroborate the importance of the C-terminal CC of CRY1 for the interaction with TIM we showed that HA-CRY1WT, but not HA-CRY1dCC, was able to translocate TIM(1–1079)-GFP from the cytoplasm to the nucleus (Fig. 5D).

Given the above interactions, we tested by Western blot how TIM behaves in the absence of CRY proteins. For that we probed with anti-TIM antibodies protein lysates of thymus (Fig. S3A), liver and spleen (Fig. S3B) obtained from Cry1−/−Cry2−/− mice which were sacrificed around the clock. We could not detect differences of TIM protein expression in absence of CRY proteins. andthe sub-cellular localization of TIM and Tim mRNA level were normal in mouse embryonic fibroblast (MEFs) derived from Cry1−/−Cry2−/− mice (Fig S3C and S3D).

Taken together, the co-immunoprecipitation and sub cellular localization experiments indicate that the N-terminus of TIM (1–309) associates with the CC embedded in the C-terminal tail of CRY1. The latter protein region critically distinguishes mammalian from insect CRYs.

### Differential affinity of TIM and PER2 for binding to CRY1

**Figure 6:**
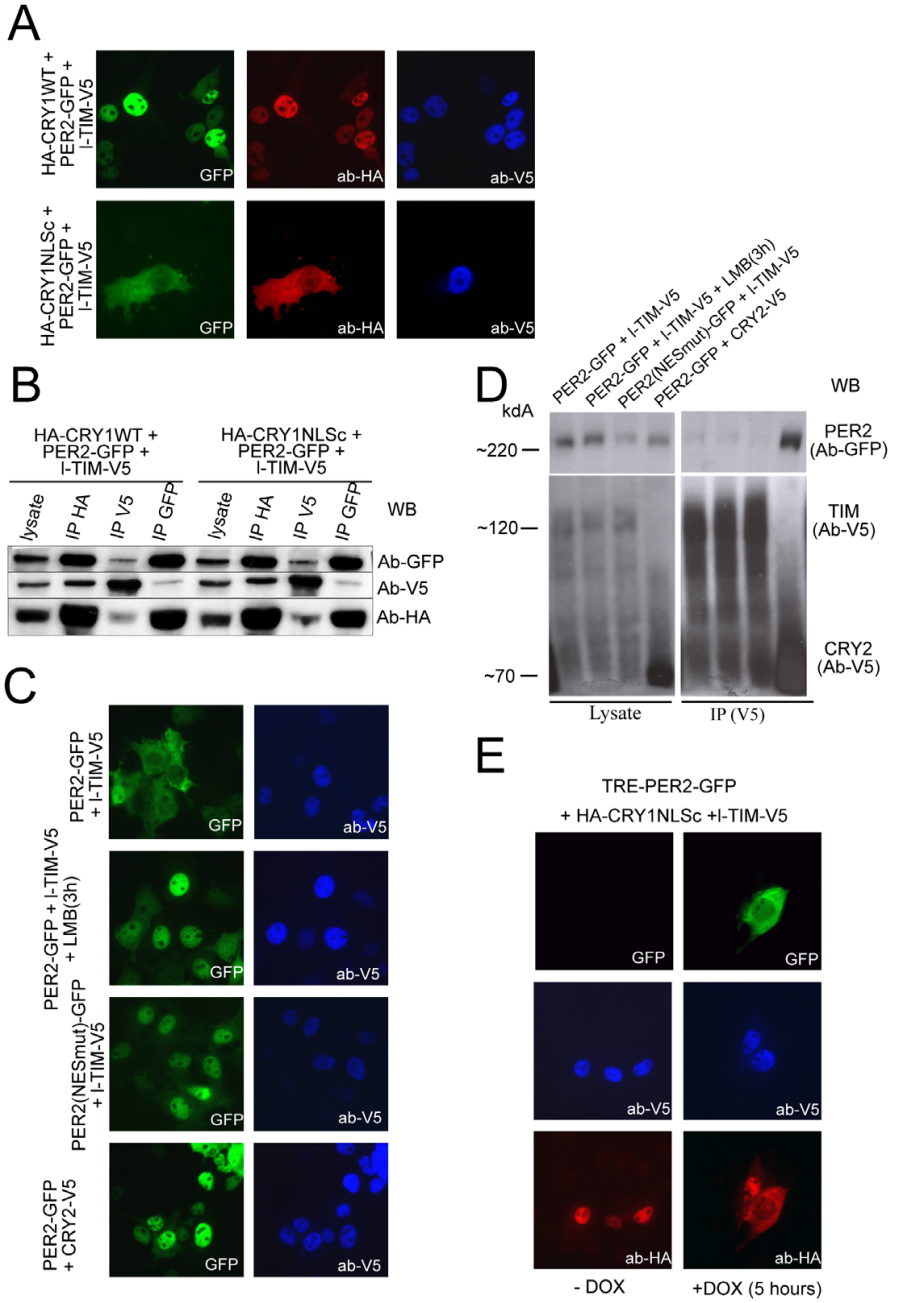
PER2 competes with TIM for binding to CRY1. **A**) Representative triple (immuno)fluorescence images of COS7 cells transiently expressing PER2-GFP, HA-CRY1 WT and l-TIM-V5 (top panels, from left to right), or HA-CRY1mutNLSc (bottom panels). Subcellular localization of proteins was visualized by fluorescence (GFP, green) or immunostaining with anti-AH (CRY1, red) or anti V5 (TIM, blue) antibodies. **B**) Characterization of CRY, PER, TIM interactions. Immunoprecipitation of either HA-CRY1 (WT or mutant NLSc using anti-HA antibodies), l-TIM-V5 (using anti-V5 antibodies) or PER2-GFP (using anti-GFP antibodies) from lysates of the cells used in panel A. The input (lysate) and precipitates were analyzed for the presence of the co-precipitated proteins using the corresponding antibodies. **C**) Representative immunofluorescence images of COS7 cells, transiently coexpressing combinations of PER2-GFP, PER2NESmut1-GFP, l-TIM-V5 and/or CRY2-V5 (as indicated in the figure) in the absence or presence of LMB. **D**) Characterization of CRY, PER, TIM interactions. Immunoprecipitation of TIM with anti-V5 antibodies from lysates of COS7 cells co-expressing: l-TIM-V5 and PER2-EGFP (in the presence or absence of LMB) or PER2NESmut-GFP. Interaction between CRY2-V5 and PER2-GFP was used as positive control for the co-immunoprecipitation procedure. The total lysates (left panel) and precipitates (right panel) were analyzed for the presence of co-precipitating proteins using the correspondent antibodies **E**) Representative immunofluorescence images of PK15 Tet-on cells co-transfected with a Dox inducible PER2 plasmid (TRE-PER2-EGFP), l-TIM-V5 and HA-CRY1mutNLSc. Cells were co-stained with anti-HA (red) and anti-V5 (blue) antibodies, before and after (5 hours) induction of PER2-EGFP expression with tetracycline (Dox).

To study the TIM-CRY-PER protein complex in mammals, we co-expressed the three differentially tagged mammalian proteins (HA-CRY1 WT, l-TIM-V5 and PER2-GFP) in COS7 cells. As shown by immuno-fluorescence experiments in Fig. 6A (upper panels), all three proteins completely co-localize into the nucleus of paraformaldehyde fixed cells. However, when cell lysates of these cultures were used to immunoprecipitate the individual over-expressed proteins, we observed that CRY1 preferentially associates with PER2 (and vice versa), and that l-TIM-V5 pull down does not result in efficient co-precipation of CRY1 or PER2 (Fig. 6B). The above results strongly suggest that in presence of PER2, TIM no longer associates with CRY1.

To further confirm that PER2 prevents TIM-CRY1 interactions, and thereby formation of CRY-PER-TIM protein complex, we replaced HA-CRY1WT in the aforementioned experiment by HA-CRY1mutNLSc. From our previous work we know that HA-CRY1mutNLSc and PER2-GFP form a complex that is in continuous nucleocytoplasmic shuttling due to the lack of a functional C-terminal NLS in CRY1 [32]. Interestingly, we observed CRY1mutNLSc and PER2 in the cytoplasm, whereas in the same cell TIM remained in the nucleus (Fig. 6A, bottom panels). The nuclear localization of TIM and the fact that this protein does not change the nucleocytoplasmic equilibrium of the CRY1mutNLSc/PER2 complex, further underlines that absence of any CRY1-PER2-TIM complexes. Subsequent co-immunoprecipitation studies confirmed that HA-CRY1mutNLSc is mainly in complex with PER2 (and vice versa), and that TIM behaves as an individual protein (Fig. 6B, right panel).

Although the above results indicate that CRY-PER-TIM complexes do not occur in mammalian cells, they do not exclude latent PER2-TIM interactions in the absence of CRY. We therefore also overexpressed l-TIM-V5 together with PER2-GFP and observed that TIM was exclusively nuclear, while PER2 displayed a nucleocytoplasmic distribution (Fig. 6C; upper panel). In line with this finding, PER2 did not substantially co-precipitate with TIM (Fig. 6D), suggesting that TIM does not bind PER2. Even forced nuclear accumulation of PER2 by culturing the cells in the presence of LMB (preventing nuclear export of NES containing proteins) or by substituting PER2-GFP for PER2NES1,2,3mut (containing three mutagenized NES sequences [10]) (Fig. 6C) did not further promote TIM and PER2 interactions, as determined by a pull down assay (Fig. 6D). As a positive control for this anti-V5 pull down experiment, we detected the expected interaction between V5-CRY2 and PER2-GFP (Fig. 6C bottom panel, and 6D).

Since we reported earlier that PER2 and PER1 associate with the C-terminal CC of CRY1 [32], the present results are all consistent with a competition between PER2 and TIM for binding to this CRY1 domain, which apparently has a higher affinity for PER2 than for TIM. To visualize this competitive process in a more dynamic way, we co-expressed HA-CRY1ΔNLSc and l-TIM-V5 together with a tetracyclin inducible vector (TRE-PER2-GFP) to control the expression of PER2-GFP. In line with the data presented in Fig. 5C, in the absence of Dox (no PER2-GFP expression) HA-CRY1ΔNLSc is translocated to the nucleus by l-TIM-V5 (Fig. 6E, left panels). In contrast, after activation of PER2-GFP expression by Dox, HA-CRY1ΔNLSc is found in the cytoplasm in complex with PER2-GFP (Fig. 6E, right panels), whereas l-TIM-V5 remains in the nucleus. Therefore, these results demonstrated that the dynamic exchange from TIM-CRY1 to PER2-CRY1 dimer can occur in the nuclear compartment.

## Discussion

In this report, we provide evidence that support a role for mammalian TIM in clock speed and resetting. Down-regulation of this gene by RNAi in both human and mouse cultured cells revealed a dual circadian phenotype: (i) shortening of circadian period by ∼1 hour; (ii) attenuated DNA damage-dependent phase advancing. To get more insight on this phenotype, we performed a detailed molecular characterization of TIM interactions with the core clock protein CRY1 and the DNA damage signal transducer CHK1, and found that the N-terminus of TIM is required for association with both proteins, as well as for homodimerization. The extreme C-terminus of TIM is instead required for its nuclear localization. Furthermore, we showed that TIM does not interact with PER2, while conversely, PER2, a clock partner of CRY1, has the potential to negatively regulate the formation of the TIM-CRY1 complex through affinity binding competition with TIM.

### TIM and the core clock

Using fibroblasts derived from Cry-deficient mice, we have proposed that the peripheral oscillator resembles the master oscillator in the SCN for key features such as the phase of clock mRNAs and the control of period length [28]. Thus, we were intrigued by the fact that the circadian phenotype observed after RNAi down-regulation of TIM in cultured cells (short period) is not comparable with that obtained by Barnes *et al*. in SCN slices (arrythmicity) [26]. Here we have convincingly shown that TIM is expressed at much higher levels in tissues undergoing proliferation (eg. spleen, thymus) than in those more differentiated such as liver. Therefore, it is conceivable that, after exposure to RNAi, residual amounts of TIM could be still present in cultured cells but not in SCN slices, and this would consequently lead to a more severe clock phenotype in the latter system. Alternatively, TIM itself, or proteins assembled with it, could cross-talk differentially with the clock in central (SCN) and peripheral organs, resulting in different circadian phenotypes after TIM down-regulation. Noteworthy, differential properties of the clock protein between central and peripheral clocks have been previously reported, although inactivation of Cry1 and Per1 genes caused a more severe phenotype in liver and cultured fibroblasts than in the central pacemaker [33].

CRY, PER and TIM are conserved players of the clock machinery in various organisms, although with the exception of CRY, their structure-function evolution is not completely understood. We decided to give a novel twist to this research area by observing these proteins from the point of view of their reciprocal interactions. Firstly, we noticed that within the C-terminal regions of CRY1/CRY2 and PER2/PER1, which have been shown to engage in reciprocal interaction, are present significant coiled coil domains (CC), whereas these domains are surprisingly absent in the C-termini of the Drosophila hortologues dCRY and dPER (Fig. S4). Secondly, it is well known that dTIM acts as a physical bridge between the light sensor dCRY and the transcriptional effector dPER by interacting with both proteins [18], whereas based on the present results mammalian TIM associates solely with CRYs (Fig. 5). PER2 appears instead to abolish the formation of the TIM/CRY1 dimer in favour of PER2/CRY1, through affinity binding competition to the C-terminal CC of CRY1 (Fig. 6). Finally, the short C-terminal TIM isoform (s-TIM-V5) does not interact with CRY1, and it has been observed that s-TIM does not undergo circadian oscillation in the SCN, whereas l-TIM does it [26]. Using coil prediction programs, we observed three major potential coiled-coil domains in TIM, located at position 237–280 (CC1), 649–695 (CC2) and 1083–1110 (CC3). Given the observed physical interaction between TIM(1–309)-GFP and HA-CRY1, we consider CC1 the most likely candidate domain for binding with CRY proteins (Fig. S4). Interestingly, such a coil coiled structure is evident in the *Drosophila* ortholog dTIM2, but less clear in dTIM, thereby supporting the previous amino acid sequence-based conclusion that TIM is the paralog of dTIM2 rather than dTIM [19].

Taken together, these observations suggest a striking evolutionary change in the mechanism of PER-CRY-TIM complex formation and provide the basis to understand it at the molecular level. We hypothesize that the evolutionary acquirement of the C-terminal CC into CRY1 and CRY2 changed these proteins from being a light sensor in Drosophila into a protein sensor in mammals. In other words, the CC of CRY would act like a “hub” for the selective and competitive docking of PER1, PER2, BMAL1, and TIM, and this interaction would lead their post-translational modifications, change in localizations and stability, and eventually become important for their properties during clock performance (Fig. S5A).

### TIM and DNA damage resetting of the clock

This dynamic interaction of proteins around the CC of CRYs could have mechanistic implications not only for the determination of period length, but also for light-independent resetting mechanism of the mammalian clock, such as that observed after DNA damage (Fig. S5B). Since we showed that the N-terminus of TIM is sufficient for the interaction with CRY1, CHK1, and with itself, it appears that this protein region may have the potential for dynamic and diversified functions that are required in both DNA damage response and clock. The fact that down-regulation of TIM attenuated the DNA damage-dependent phase advance in cultured cells confirms our hypothesis and indicate TIM as an excellent candidate to bridge those two pathways post-translationally. Noteworthy, the interaction between TIM and CHK1 increases after exposure to DNA damage [23], and therefore future experiments will tell whether CHK1 and CRY1 might compete for binding to TIM, and if over-expression and/or inactivation of CRY1 would affect DNA-damage dependent phase advance. A recent paper showed that CRYs interact in the cytoplasm with GPCR, thereby modulating the gluconeogenesis program [34]. It is tempting to speculate that a similar competitive interaction mechanism, as observed here between TIM and PER2, may also occur at certain timing between PER2 and GPCR for binding to the CC of CRY1, resulting in the release of CRY1 from GPCR-mediated cytoplasmic retention in favour of PER-mediated nuclear translocation.

### TIM in proliferative tissues

In this study we could detect a circadian expression of TIM in the intestine, where it co-localizes with the proliferative marker Ki67 at the base of the intestinal crypt, showing peaking levels at ZT4 and ZT8. Notably, S phase in this tissue is mainly occurring at ZT5 *in vivo* [35] and TIM expression is selectively detected during the S/G2/M phases of the cell cycle in cultured cells [23]. This would also explain the extremely low levels of TIM observed in the liver, a tissue containing most cells at the G0/G1 state. Since we could neither detect circadian variations of TIM expression in spleen and thymus, nor a CRY-dependent expression pattern, TIM oscillation in the intestine may simply represents a circadian-dependent cell cycle synchronization at the systemic level, rather than a cell autonomous mechanism. In support of this hypothesis we showed that TIM is normally expressed in MEF's derived from Cry1−/−Cry2−/− and, more importantly, in the thymus and spleen of Cry1−/−Cry2−/− mice, indicating that under basal lightening conditions (LD) its regulation is CRY-independent in proliferative peripheral clock tissues.

Ultimately, to understand whether TIM has the same clock function in proliferative (intestine, spleen) and non-proliferative peripheral tissues (liver, kidney), as well as SCN, tissue-specific inactivation of *mTim* in those organs will be required.

## Materials and Methods

### Ethics statement

Mice were kept at the Animal Resource Center (Erasmus University Medical Center), which operates in compliance with European guidelines (European Community 1986) and The Netherlands legislation for the protection of animals used for research, including ethical review. Animal studies at Erasmus University Medical Center were approved by DEC Consult, an independent Animal Ethical Committee (Dutch equivalent of the IACUC) under permit numbers 139-09-02 (EUR1702), 139-09-11 (EUR1760) and 139-09-12(EUR1761)

### Plasmids

To express full length mouse TIM, we used TIM(1–1198)-V5 (l-TIM-V5), cloned in the pcDNA3.1 vector (a kind gift from S Reppert). To express the short isoform of TIM, we recloned a 2.5 kb NcoI fragment, encoding the C-terminal part of TIM, including the V5 tag and stop codon, in pcDNA3.1 Hygro. This DNA fragment contains 12 extra nucleotides upstream the ATG2 at amino acid position 732. Since we were able to detect clear expression of the resulting protein using a V5 antibody, we concluded that the ATG at position 732 is capable to provide the first Methionine and engage in translation to produce the short TIM isoform. The expression vectors TIM(1–309)-GFP and TIM(1–1079)-GFP were generated by recloning the HindIII-BglII and HindIII-EcoRI fragments from TIM(1–1198)-V5 in pEGFP-N1 (Clontech). GFP-TIM(1079–1198) was generated by recloning the EcoRI-ApaI fragment from TIM(1–1198)-V5 in pEGFP-C3 (Clontech). HA-CRY1 and PER2-GFP plasmids have been previously described [32].PER2-GFP-NESmut, TRE-PER2-EGFP and CRY2-V5 were provided by K. Yagita and Flag-CHK1 by Jiri Bartek (Institute of Cancer Biology and Centre for Genotoxic Stress Research, Danish Cancer Society, Denmark).

### Lentiviral short hairpin RNA (shRNA)

To knock down the expression of murine *Tim* we used a successfully validated shRNA expressing lentiviral vector (TCRN0000097989 cl.2210 from Sigma library) [29], as well as in house made pSuper vector targeting the sequence ATGCAGTTGCTGAAACAA (shRNA#4). To interfere with the expression of human *TIM*, we made use of the lentiviral vectors from Sigma library TRCN0000153090 (cl.2267), TRCN0000153760 (cl.2268), TRCN0000157650 (cl.2270); Control shRNA vectors employed were SHC002 (cl.153). All shRNA downregulation experiments were performed in parallel with a negative control.

### Cell culture and transfection

COS7, NIH 3T3 (American Type Culture Collection), and HEK293T (American Type Culture Collection) cells, as well as wild type and *Cry1^−/−^/Cry2^−/−^* primary dermal fibroblasts (MDFs) were cultured in Dulbecco's modified Eagle's medium-F10-Pen/Strep-10% fetal calf serum. The porcine kidney PK15 Tet-inducible cell line has been previously described [36]. Transient expression studies were performed by transfecting cells with plasmids using Fugene reagent (Boehringer) according to the manufacturer's instructions. For luminescence measurements Per2::Luciferase (Per2-Luc) and pGl4.11-Bmal1::luciferase (Bmal1-Luc) (kindly provided by Dr. U. Schibler, Geneva) was used as a reporter. Leptomycin treatment missing (LMB was added for 3 hours before immunostaining)

### Real time bioluminescence monitoring and ionizing radiation mediate phase shift

To monitor circadian oscillations in cell cultures in real time, cells were cultured in medium buffered with 25 mM HEPES and containing 0.1 mM luciferin (Sigma). After synchronization of intracellular clocks by treatment of confluent cultures with forskolin (dissolved in 100% ethanol, added to the culture medium at a final concentration of 30 µM), bioluminescence was recorded for 7 days (75 sec measurements at 10 min intervals) with a LumiCycle 32-channel automated luminometer (Actimetrics) placed in a dry, temperature-controlled incubator at 37°C. Data was analysed with the Actimetrics software and two sample comparisons were done using a Students T-test.

Ionizing radiation exposure was performed as described previously [14]. Briefly, confluent culture dishes where placed in a ^137^Cs γ-radiation source approximatively 28 hour after synchronization (corresponding to the lowest level of Bmal1-Luc). Mock-treated cells (culture dishes having been subjected to exactly the same procedure except that γ -radiation was omitted) served as an internal control.

### Co-immunoprecipititon and immunofluorescence experiments

Co-immunoprecipitation studies were performed as described previously [32]. In short, we transiently expressed the plasmids described above in COS7 cells and used anti-FLAG antibodies (Sigma), or anti-HA, or V5, antibodies for the immunoprecipitation, immunoblot and immunofluorescence analysis step (1∶1000 dilution). As secondary antibody, we used horseradish peroxidase conjugated anti-mouse IgG (DAKO) and anti-rabbit IgG (BioSource), and corresponding fluorescein-conjugated antibodies, at a 1∶1000 dilution Chemoluminescence was detected using the ECL system (Pharmacia Biotech). Western blots were performed with an anti-TIM [37] and anti-CRY1 antibodies generously donated by Dr. P. Minoo and Dr. J.A. Ripperger, respectively.

## Supporting Information

Figure S1 **Verification of mTIM and hTIM downregulation by shRNA.** **A**) WB analysis for TIM expression in a panel of equally loaded amounts of adult mouse tissues lysates (Kidney K, Spleen Sp, Liver L, Testis T). The replica filters were probed with two independent anti-TIM antibodies (one from P. Minoo above [37], and M19 from Santa Cruz in the middle), which gave the same pattern, and also detected the same TIM band in NIH3T3 lysates (data not shown). A background band was used as loading control (bottom). All subsequent expression analysis of TIM was performed with antibodies from P. Minoo. **B**) Western blot analysis of protein lysates derived from NIH 3T3 cells 48 hour after transient transfection with independent pSuper shRNA constructs directed against mouse *Tim* (shRNA#1 to shRNA#4), or a non targeting sequence (shRNActr.). Untreated represents untransfected cells, equal amounts of protein lysates were loaded. The filter was probed with anti-TIM antibodies and with anti-Actin antibodies as loading control. **C**) WB analysis of protein lysates derived from HEK293 cells co-transfected with GFP (transfection control), l-TIM-V5, and either shRNActr or shRNA#4. The filter was probed with anti-V5 and anti-GFP antibodies. **D**) Immunofluorescence of NIH/3T3 co-transfected with mitochondrial localized GFP (green) and shRNActr. (left), or shRNA#4 (right). After 48 hours cells were fixed and endogenous TIM was detected in GFP-positive cells with anti-TIM antibodies (red). Nuclei are counterstained with DAPI (blue). **E**) qPCR quantification of hTim mRNA downregulation using 3 independent shRNA constructs against hTim presented in Fig. 3F. As internal control the expression of hTim was measured in presence of non-targeting shRNA (clone 153).
(TIF)

Figure S2 **TIM co-immunoprecipitates with HA-CRY1 and Flag-CHK1.** **A**) Immunofluorescence aanalysis of NIH 3T3^HA-CRY1^ cells stably expressing HA-CRY1 WT from a CMV promoter. Fixed cells were double stained with rat anti-HA (green) and rabbit anti-TIM (red) antibodies. **B**) The lysates from NIH3T3 (plain) and NIH 3T3^HA-CRY1^ cells were subjected to immunoprecipitation with anti-HA antibodies (NIH 3T3^HA-CRY1^ cells buffer contained increasing amounts of TritonX, with the maximum levels also being used for NIH3T3 plain). The upper panel shows an immunoblot of total cell lysates (input) revealing the presence of endogenous TIM in all samples (HA-CRY1 is not extracted in sample 2 containing low concentrations of TritonX). After immuneprecipitation (IP-HA) the filter was subsequently probed with anti-HA and anti-TIM antibodies. Specificity of TIM co-immunoprecipitation is shown by the negative staining after pull down experiment with normal NIH 3T3 cells (plain). **C**) Identification of the CHK1 binding region in TIM. HEK293 cells were transfected with plasmids expressing Flag-CHK1 and various combination of TIM deletion constructs. Total lysates were prepared and subjected to immunoprecipitation using anti-Flag antibody (right panel). Immunoprecipitated proteins were detected by Western blot analysis using indicated antibodies (anti-V5 and anti-GFP). Input is shown in the left panel.
(TIF)

Figure S3 **TIM expression is not affected by lack of CRY1 and CRY2 in proliferative tissues.** **A**) Western blot analysis of temporal TIM expression in the thymus of *Cry1^−/−^/Cry2^−/−^* mice sacrificed around the clock. **B**) Western blot analysis of TIM expression in adult liver (top) and spleen (bottom) from wild type (WT) and *Cry1^−/−^/Cry2^−/−^* mice, housed under a LD12∶12 light regime. Samples were collected at two critical time points (ZT8 and ZT20. β-Actin immunostaining served as a loading and control, while CRY immunostaining confoirmed the genetic status of the mice. **C**) Representative immunofluorescence pictures of proliferating WT and *Cry1^−/−^/Cry2^−/−^* MEFs. showing that endogenous TIM is normally detected in the nuclei. **D**) Quantification of *Per2* and *Tim* mRNA expression in proliferative wild type (WT, blue) and *Cry1^−/−^/Cry2^−/−^* (red) primary MEFs. The Y axis shows the raw values derived from a microarray experiment. Whereas Per2 is upregulated in *Cry1^−/−^/Cry2^−/−^* MEFs due to lost of CRY repression (used as positive control of this type of measurement), expression of Tim is not affected.
(TIF)

Figure S4 **Coiled-coil domains in** ***Drosophila*** **and mammalian clock proteins.** Comparative coiled-coil analysis for CRY, PER and TIM proteins in *Drosophila* and mammals. The yellow stars indicate coiled-coil domains present in mammalian CRYs and PERs (but not in *Drosophila*) that engaged in interactions as previously reported [10], [32] and the TIM region interacting with CRY1 and CHK1 described this manuscript.
(TIF)

Figure S5 **A model for the assembly of core clock proteins in which TIM may play a dual role.** **A**) In Drosophila dCRY is a pure light sensor and dTIM helps to transmit the light information to the clock machinery by interacting with both dCRY and dPER. Notably, dCRY does not interact with dPER. By contrast, in mammals CRY interacts with most of the core clock proteins (PER1, PER2, CLOCK, BMAL1, TIM) and therefore it is at the center of the negative transcription loop. We identified a versatile molecular surface of the CRY protein, rappresented by the C-terminal coiled coil domain (CC), which mediates interaction with PER2, but it is used also for interaction with TIM or BMAL1. These CRY partners are in competition with each others, possibly changing the stochiometry and function of the clock machinery in time, or the way it perceives external stimuli. By contrast, in Drosophila changes in clock stochiometry are strongly under light regulation, which triggers CRY and TIM degradation. In addition, NLS and NES present in almost all clock proteins add another level of complexity to these post-translational mechanisms. While it has been shown that dTIM undergoes nucleocytoplasmic shuttling through a well characterized NES, it is unknown if TIM also carries a functional NES. **B**) In mammals TIM may perform a bridge function between the ATM/ATR/CHK1 pathway that senses DNA damage and the core clock through CRY association, thereby allowing clock phase advance to occur through a yet unknown mechanism. On the other hand, the same DNA damage signal can be transmitted to the clock through the well-established association of ATM with PER2.
(TIF)
