# Supplementary figures and images for: Mammalian TIMELESS Is Involved in Period Determination and DNA Damage-Dependent Phase Advancing of the Circadian Clock
Source: PLoS One. 2013 Feb 13;8(2):e56623. doi: 10.1371/journal.pone.0056623 (PMC3572085; doi:10.1371/journal.pone.0056623)

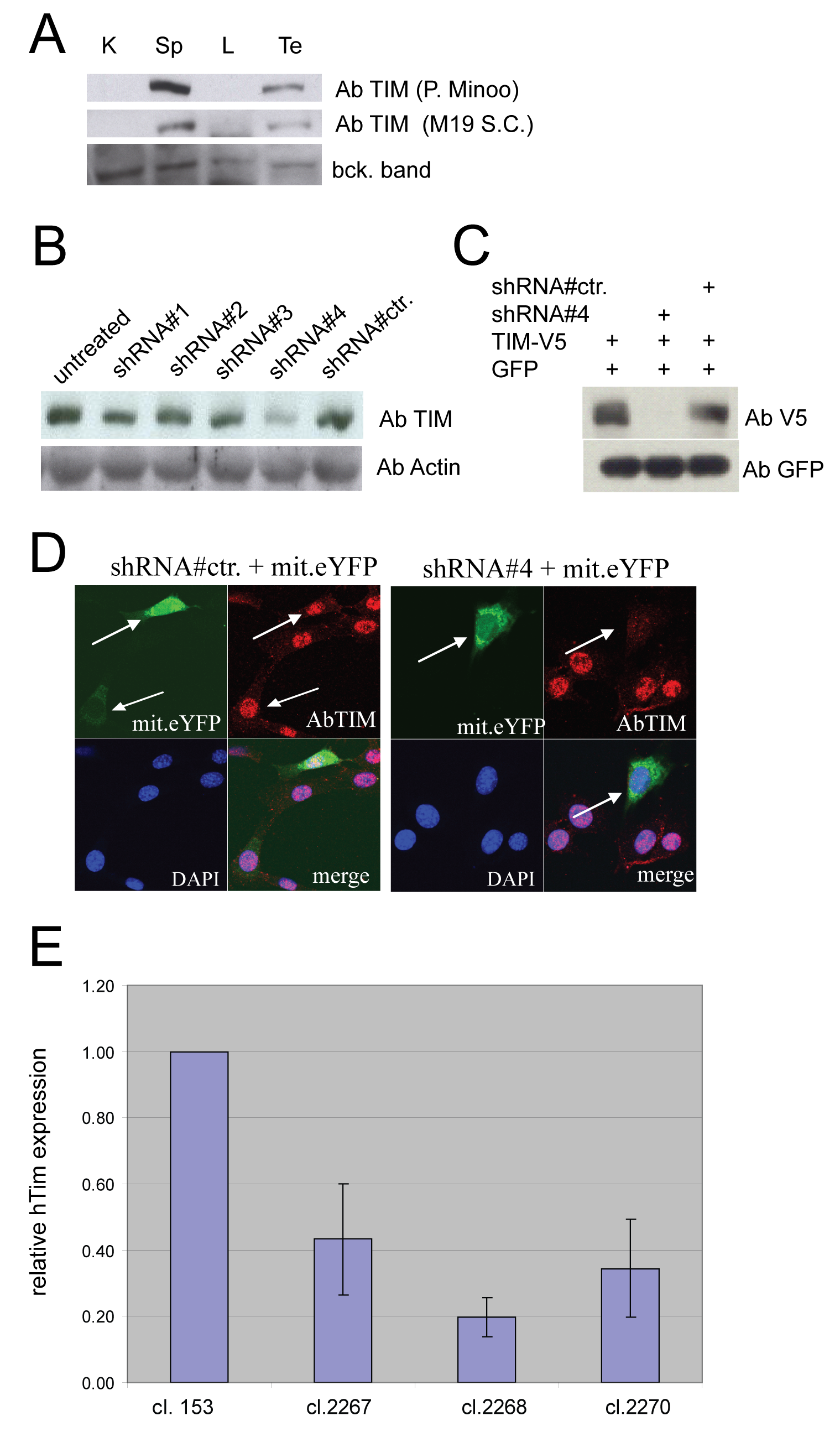

Supplement: Figure S1 — Verification of mTIM and hTIM downregulation by shRNA. A) WB analysis for TIM expression in a panel of equally loaded amounts of adult mouse tissues lysates (Kidney K, Spleen Sp, Liver L, Testis T). The replica filters were probed with two independent anti-TIM antibodies (one from P. Minoo above [37], and M19 from Santa Cruz in the middle), which gave the same pattern, and also detected the same TIM band in NIH3T3 lysates (data not shown). A background band was used as loading control (bottom). All subsequent expression analysis of TIM was performed with antibodies from P. Minoo. B) Western blot analysis of protein lysates derived from NIH 3T3 cells 48 hour after transient transfection with independent pSuper shRNA constructs directed against mouse Tim (shRNA#1 to shRNA#4), or a non targeting sequence (shRNActr.). Untreated represents untransfected cells, equal amounts of protein lysates were loaded. The filter was probed with anti-TIM antibodies and with anti-Actin antibodies as loading control. C) WB analysis of protein lysates derived from HEK293 cells co-transfected with GFP (transfection control), l-TIM-V5, and either shRNActr or shRNA#4. The filter was probed with anti-V5 and anti-GFP antibodies. D) Immunofluorescence of NIH/3T3 co-transfected with mitochondrial localized GFP (green) and shRNActr. (left), or shRNA#4 (right). After 48 hours cells were fixed and endogenous TIM was detected in GFP-positive cells with anti-TIM antibodies (red). Nuclei are counterstained with DAPI (blue). E) qPCR quantification of hTim mRNA downregulation using 3 independent shRNA constructs against hTim presented in Fig. 3F. As internal control the expression of hTim was measured in presence of non-targeting shRNA (clone 153). (TIF) [file pone.0056623.s001.tif]

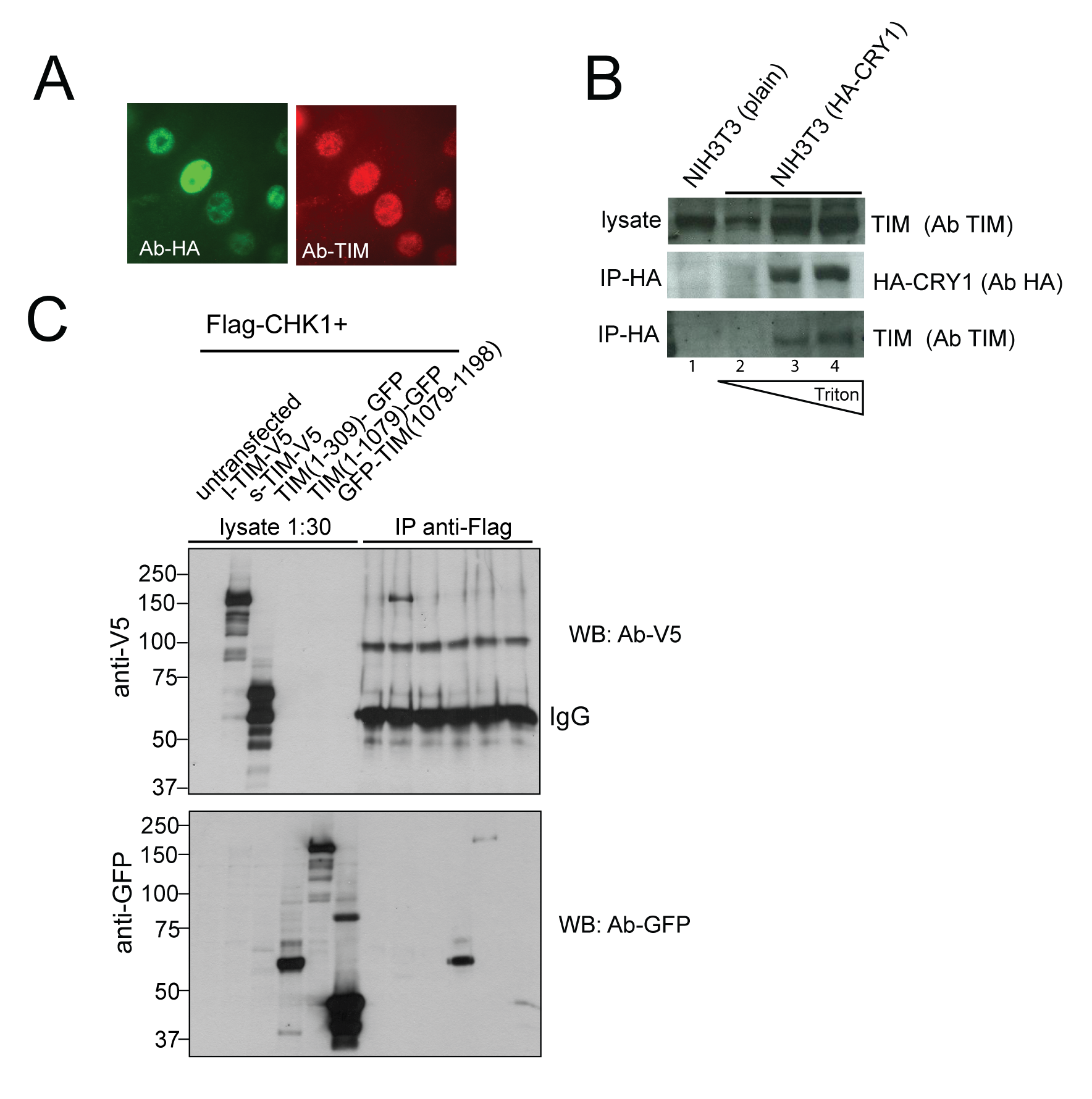

Supplement: Figure S2 — TIM co-immunoprecipitates with HA-CRY1 and Flag-CHK1. A) Immunofluorescence aanalysis of NIH 3T3HA-CRY1 cells stably expressing HA-CRY1 WT from a CMV promoter. Fixed cells were double stained with rat anti-HA (green) and rabbit anti-TIM (red) antibodies. B) The lysates from NIH3T3 (plain) and NIH 3T3HA-CRY1 cells were subjected to immunoprecipitation with anti-HA antibodies (NIH 3T3HA-CRY1 cells buffer contained increasing amounts of TritonX, with the maximum levels also being used for NIH3T3 plain). The upper panel shows an immunoblot of total cell lysates (input) revealing the presence of endogenous TIM in all samples (HA-CRY1 is not extracted in sample 2 containing low concentrations of TritonX). After immuneprecipitation (IP-HA) the filter was subsequently probed with anti-HA and anti-TIM antibodies. Specificity of TIM co-immunoprecipitation is shown by the negative staining after pull down experiment with normal NIH 3T3 cells (plain). C) Identification of the CHK1 binding region in TIM. HEK293 cells were transfected with plasmids expressing Flag-CHK1 and various combination of TIM deletion constructs. Total lysates were prepared and subjected to immunoprecipitation using anti-Flag antibody (right panel). Immunoprecipitated proteins were detected by Western blot analysis using indicated antibodies (anti-V5 and anti-GFP). Input is shown in the left panel. (TIF) [file pone.0056623.s002.tif]

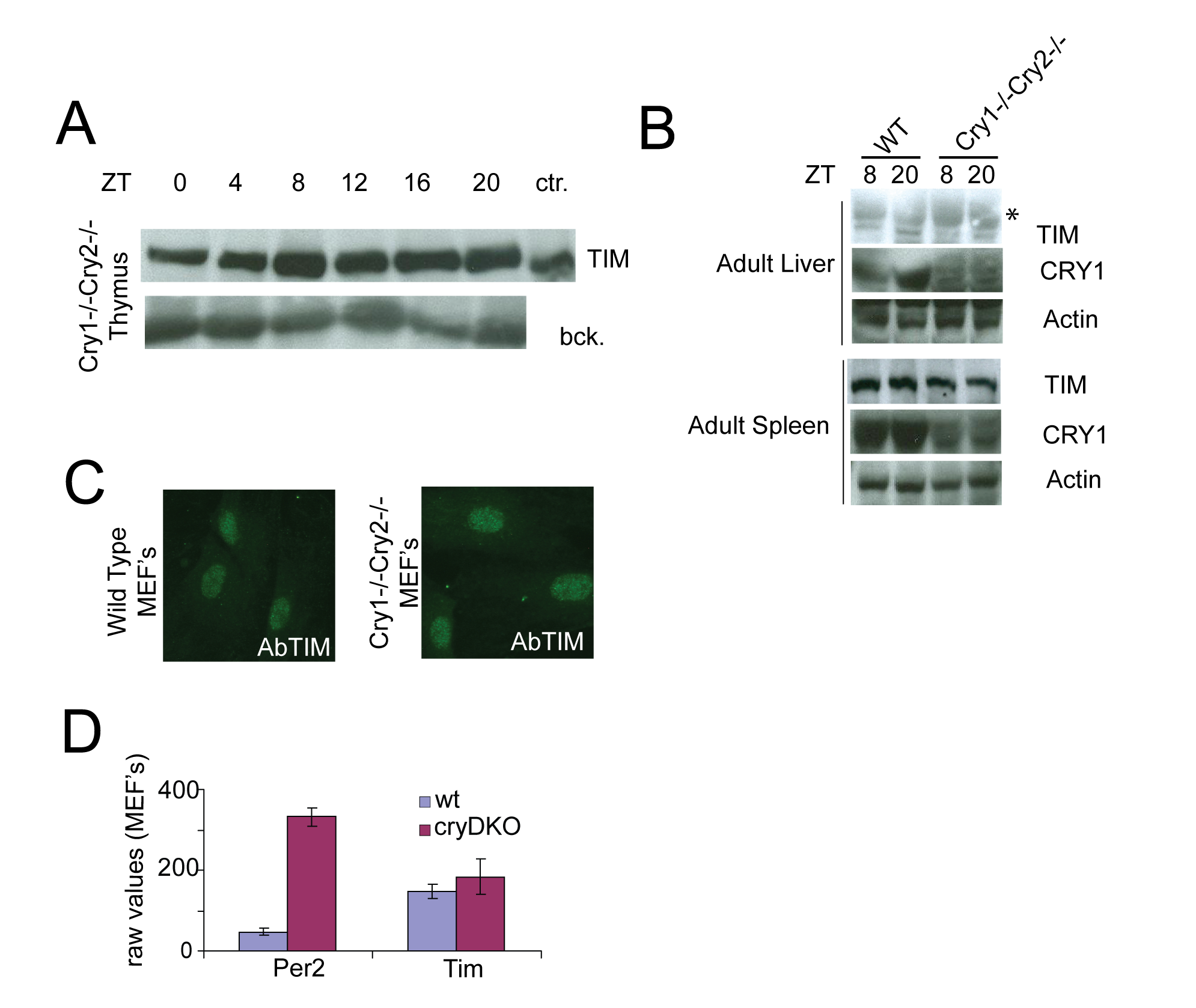

Supplement: Figure S3 — TIM expression is not affected by lack of CRY1 and CRY2 in proliferative tissues. A) Western blot analysis of temporal TIM expression in the thymus of Cry1−/−/Cry2−/− mice sacrificed around the clock. B) Western blot analysis of TIM expression in adult liver (top) and spleen (bottom) from wild type (WT) and Cry1−/−/Cry2−/− mice, housed under a LD12∶12 light regime. Samples were collected at two critical time points (ZT8 and ZT20. β-Actin immunostaining served as a loading and control, while CRY immunostaining confoirmed the genetic status of the mice. C) Representative immunofluorescence pictures of proliferating WT and Cry1−/−/Cry2−/− MEFs. showing that endogenous TIM is normally detected in the nuclei. D) Quantification of Per2 and Tim mRNA expression in proliferative wild type (WT, blue) and Cry1−/−/Cry2−/− (red) primary MEFs. The Y axis shows the raw values derived from a microarray experiment. Whereas Per2 is upregulated in Cry1−/−/Cry2−/− MEFs due to lost of CRY repression (used as positive control of this type of measurement), expression of Tim is not affected. (TIF) [file pone.0056623.s003.tif]

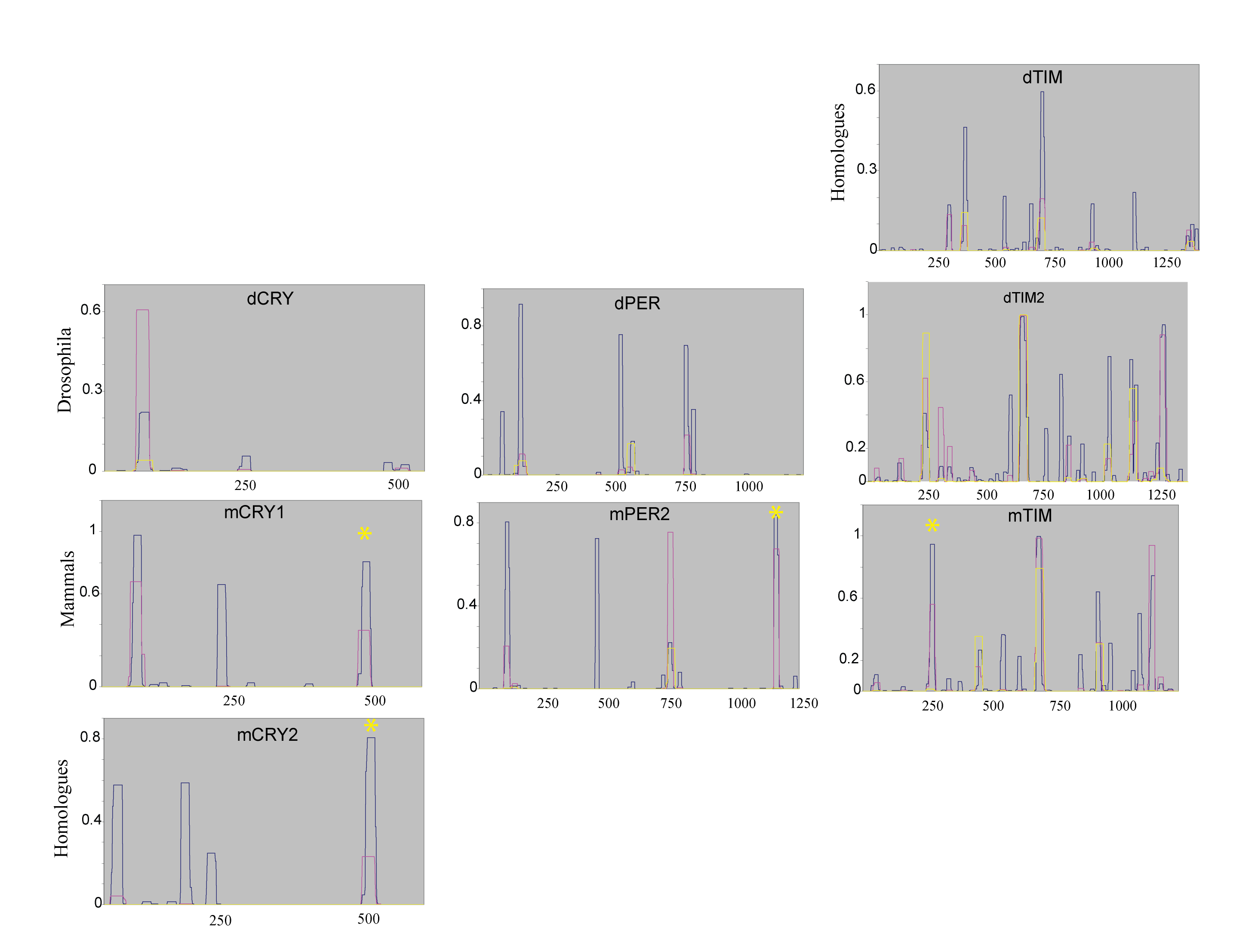

Supplement: Figure S4 — Coiled-coil domains in Drosophila and mammalian clock proteins. Comparative coiled-coil analysis for CRY, PER and TIM proteins in Drosophila and mammals. The yellow stars indicate coiled-coil domains present in mammalian CRYs and PERs (but not in Drosophila) that engaged in interactions as previously reported [10], [32] and the TIM region interacting with CRY1 and CHK1 described this manuscript. (TIF) [file pone.0056623.s004.tif]

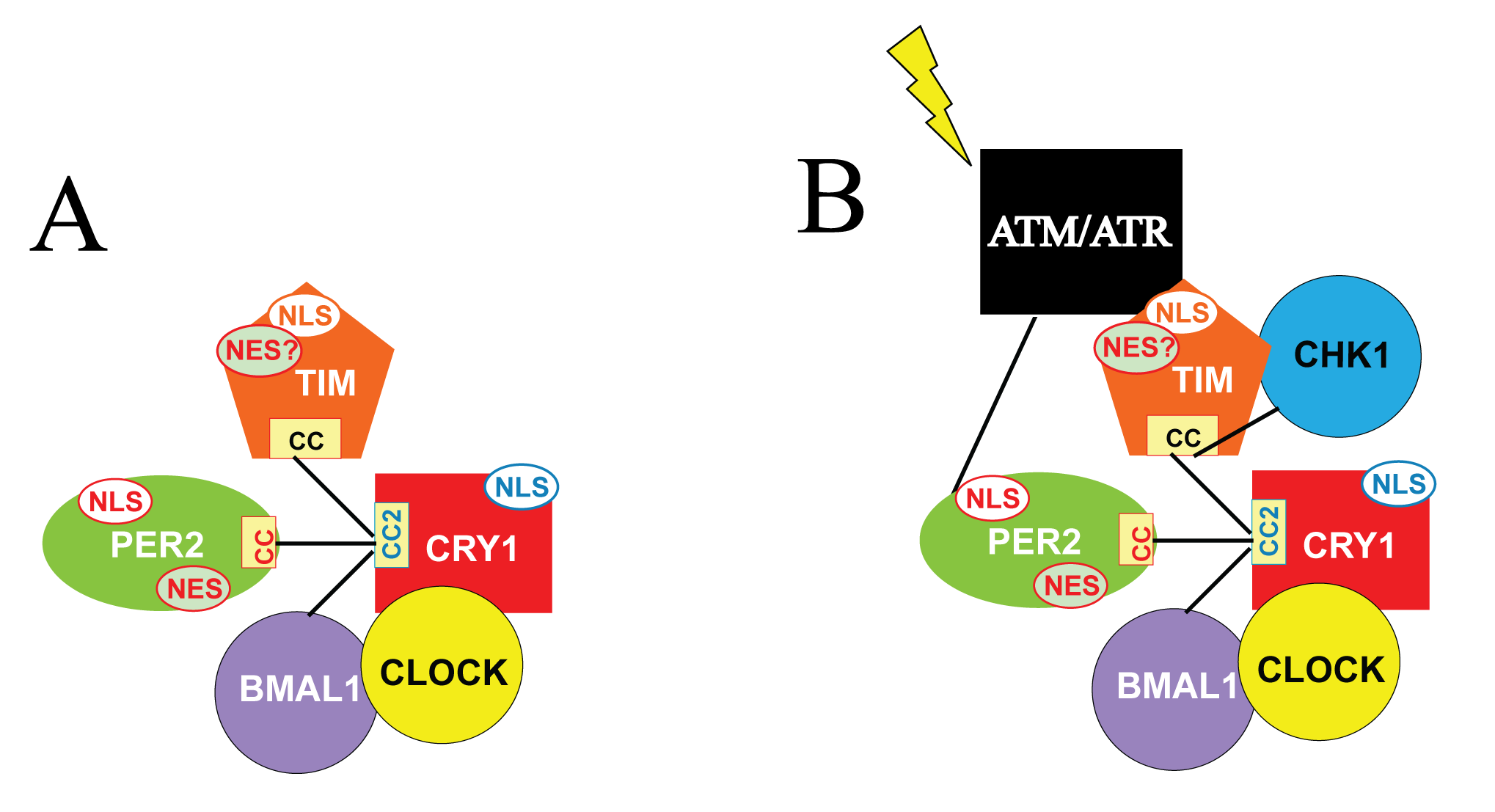

Supplement: Figure S5 — A model for the assembly of core clock proteins in which TIM may play a dual role. A) In Drosophila dCRY is a pure light sensor and dTIM helps to transmit the light information to the clock machinery by interacting with both dCRY and dPER. Notably, dCRY does not interact with dPER. By contrast, in mammals CRY interacts with most of the core clock proteins (PER1, PER2, CLOCK, BMAL1, TIM) and therefore it is at the center of the negative transcription loop. We identified a versatile molecular surface of the CRY protein, rappresented by the C-terminal coiled coil domain (CC), which mediates interaction with PER2, but it is used also for interaction with TIM or BMAL1. These CRY partners are in competition with each others, possibly changing the stochiometry and function of the clock machinery in time, or the way it perceives external stimuli. By contrast, in Drosophila changes in clock stochiometry are strongly under light regulation, which triggers CRY and TIM degradation. In addition, NLS and NES present in almost all clock proteins add another level of complexity to these post-translational mechanisms. While it has been shown that dTIM undergoes nucleocytoplasmic shuttling through a well characterized NES, it is unknown if TIM also carries a functional NES. B) In mammals TIM may perform a bridge function between the ATM/ATR/CHK1 pathway that senses DNA damage and the core clock through CRY association, thereby allowing clock phase advance to occur through a yet unknown mechanism. On the other hand, the same DNA damage signal can be transmitted to the clock through the well-established association of ATM with PER2. (TIF) [file pone.0056623.s005.tif]
